# *Plasmodium falciparum* Variant Surface Antigen Expression Patterns during Malaria

**DOI:** 10.1371/journal.ppat.0010026

**Published:** 2005-11-18

**Authors:** Peter C Bull, Matthew Berriman, Sue Kyes, Michael A Quail, Neil Hall, Moses M Kortok, Kevin Marsh, Chris I Newbold

**Affiliations:** 1 Nuffield Department of Clinical Medicine, John Radcliffe Hospital, University of Oxford, Oxford, United Kingdom; 2 Wellcome Trust/Kenya Medical Research Institute Collaborative Programme, Kilifi, Kenya; 3 Wellcome Trust Sanger Institute, Hinxton, United Kingdom; 4 The Institute for Genomic Research, Rockville, Maryland, United States of America; Harvard School of Public Health, United States of America

## Abstract

The variant surface antigens expressed on *Plasmodium falciparum*–infected erythrocytes are potentially important targets of immunity to malaria and are encoded, at least in part, by a family of *var* genes, about 60 of which are present within every parasite genome. Here we use semi-conserved regions within short *var* gene sequence “tags” to make direct comparisons of *var* gene expression in 12 clinical parasite isolates from Kenyan children. A total of 1,746 *var* clones were sequenced from genomic and cDNA and assigned to one of six sequence groups using specific sequence features. The results show the following. (1) The relative numbers of genomic clones falling in each of the sequence groups was similar between parasite isolates and corresponded well with the numbers of genes found in the genome of a single, fully sequenced parasite isolate. In contrast, the relative numbers of cDNA clones falling in each group varied considerably between isolates. (2) Expression of sequences belonging to a relatively conserved group was negatively associated with the repertoire of variant surface antigen antibodies carried by the infected child at the time of disease, whereas expression of sequences belonging to another group was associated with the parasite “rosetting” phenotype, a well established virulence determinant. Our results suggest that information on the state of the host–parasite relationship in vivo can be provided by measurements of the differential expression of different *var* groups, and need only be defined by short stretches of sequence data.

## Synopsis

Hope that it will be possible to develop a malaria vaccine is supported by the fact that individuals who have grown up in malaria endemic regions learn to carry malarial infections without suffering disease. Surprisingly little is still known about how this immunity develops. Much current research focuses on how the host develops immune responses to parasite antigens that are exposed to the host immune system. A major family of such antigens are inserted into the surface of parasite-infected erythrocytes, where they undergo antigenic switching to evade a developing antibody response. These proteins are encoded by a family of approximately 60 *var* genes, variants of which are present in every parasite genome.

The extreme diversity of the *var* genes has prevented meaningful comparison of their expression in clinical isolates. However, the authors of this paper show that *var* genes can be placed in groups that have a similar representation in the genomes of all parasites that the authors collected from Kenyan children. Having demonstrated an underlying similarity at the genomic level, the authors show that the *var* expression patterns vary markedly between different patients. The expression levels of specific groups of *var* genes was associated with poorly developed antibody responses in the children and a well-established parasite virulence phenotype. The study provides tools for exploring how host and parasite adapt to one another as immunity develops.

## Introduction

In sub-Saharan Africa, *Plasmodium falciparum* malaria infection is a major cause of childhood mortality. Adults, though still susceptible to infection, are protected against severe forms of malaria. Despite considerable attention over the last decade, this naturally acquired immunity is poorly understood at the molecular level. Even less understood is why, despite similar exposure levels, some children get severe malaria and die whereas others never succumb to life-threatening disease. Molecular tools to type infecting parasites and to give meaningful information about the host–parasite interaction in vivo are needed urgently.

*P. falciparum* erythrocyte membrane protein 1 (PfEMP1) plays a central role in the host–parasite interaction. Members of this family of molecules are inserted into the surface of infected erythrocytes by parasites during the asexual stage of growth. PfEMP1 molecules are encoded by a family of about 60 highly diverse *var* genes [1] that undergo rapid switching in vitro and are thought to be largely responsible for the well characterized phenomenon of clonal antigenic variation [2–5]. In addition, they appear to be central to changes in cytoadherence properties that lead to the sequestration of infected erythrocytes in capillary beds, potentially a key step in the pathology of severe disease [6,7]. The molecules are made up of combinations of different domains, each mediating a specific range of interactions with molecules on host endothelial cells [8–10], platelets [11], uninfected erythrocytes [12,13], and dendritic cells [14].

PfEMP1 proteins are presently the best candidates for the variant surface antigens (VSAs) proposed as targets for naturally acquired immunity to malaria [15]. Following acute disease, children develop specific immune responses to the repertoire of VSAs that caused the infection. The anti-VSA antibodies carried by the host at the time of disease impose a selection pressure on the repertoire of VSAs expressed during an infection [16–18]. Thus, naturally acquired immunity may develop through the piecemeal acquisition of a large repertoire of anti-VSA antibodies [16]. This is supported by the demonstration that PfEMP1-based vaccines provide protection against experimental infection with a specific parasite genotype [19].

**Figure 1:**
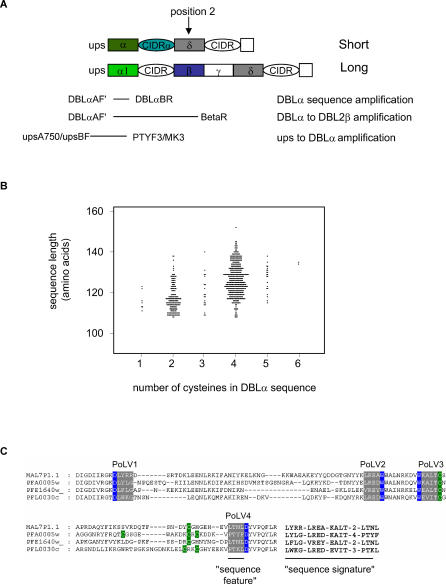
Organizational Features of *var* Genes (A) *var* gene organization. The *var* genes are complex, multi-domain structures composed of variable numbers of DBL domains (rectangles) of different sequence classes (DBLα, -β, -γ, -δ, and -ɛ plus several heterogeneous DBLX) and cysteine-rich interdomain regions (CIDR, ovals), also of different classes (CIDRα, -β, and -γ). Despite this complexity, the majority of *var* genes in the 3D7 genome can be described according to (1) whether they belong to a small set of long genes that encode PfEMP1 molecules with 6–9 domains or to a much larger set of short genes that encode short PfEMP1 molecules with only four domains; (2) their telomeric or internal position within the chromosome; (3) their direction of transcription, either towards the telomere or towards the centromere; (4) the sequence of their second most N-terminal DBL domain (DBL2), either DBL2β, DBL2δ, or DBL2γ; and (5) their association with one of five upstream (ups) regulatory sequences, upsA, upsB, upsC, upsD, or upsE [1,25,26]. The genes do not fall randomly within these categories. For example, of the long *var* genes, most have a DBL2β and are associated with upsA, whereas most short genes have a DBL2δ. Of the telomeric genes, most of those that are transcribed towards the telomere are associated with upsA, whereas all those transcribed towards the centromere are associated with upsB [28,33]. This apparent genetic structuring is associated with functional specialization. A subset of CIDR regions (CIDRα) situated immediately 3′ of DBLα bind to CD36 when expressed as recombinant peptides (teal shaded oval). These regions are generally replaced by non-CD36-binding CIDR regions in long *var* genes [27]. In 3D7, long *var* genes tend to be associated with a distinct subset of DBLα domains called “DBLα1” [27]. This observation is potentially very useful since DBLα and DBLα1 sequences can be PCR amplified from nearly all *var* genes using a universal set of degenerate primers [29]. Examples of long and short *var* genes found in the 3D7 genome are shown. A white square is used to represent the conserved exon 2 at the C-terminal end. Short genes are relatively conserved in domain structure. Long genes have variable organization. Two forms of PCR product were cloned and sequenced. DBLα-specific primers (DBLαAF′ and DBLαBR) were used to amplify DBLα sequences from cDNA and genomic DNA from each parasite isolate. DBLαAF′ and BetaR primers were used to amplify genomic DNA from isolates 4111 and 4161. Four further primers were used in different combinations to amplify between DBLα and ups. (B) Distinct DBLα sequences categorized according to the number of cysteine residues in the sequence. The number of cysteines present in each distinct DBLα sequence is plotted against the length of the sequence. (C) The location of sequence features. PoLVs are in gray. The conserved amino acids to which they are anchored are in blue. Cysteine residues are highlighted in green. The derived “sequence signature” for each clone is indicated.

PfEMP1 proteins have generally been considered to be too diverse to be of use in a malaria vaccine. This diversity appears to be generated, at least in part, by intragenic recombination between *var* genes [20,21], raising fears that it may be impossible to classify these genes in any meaningful sense. However, other observations suggest this diversity may be finite. First, VSAs expressed in children with severe malaria show evidence of having restricted diversity. Parasite isolates from children are recognised at different frequencies by plasma collected from the childhood population in the same geographical location. This frequency of recognition [17,22] is dependent on the immune status of the host, being negatively associated with host age and positively associated with disease severity [17,18,22,23]. Commonly recognised VSAs also appear to have a broad geographical distribution [24]. Second, complete genome sequencing of laboratory *P. falciparum* line 3D7 [1] revealed genetic structuring of the *var* repertoire within the genome. Different subsets of *var* genes exist that are associated with different upstream control elements [25,26] and functional properties [27,28]. Importantly, differences in the functional properties of the proteins appear to be reflected in the sequence of the Duffy-binding-like (DBL) α domain, the only *var* domain that is PCR amplifiable from nearly all *var* genes (See Figure 1A for more details).

A key question is whether the genetic structuring observed in 3D7 is universal enough to allow the development of a biologically meaningful *var* gene typing system. Here we have addressed this question using large-scale sequencing of short *var* sequence tags from the DBLα domain of genomic DNA and expressed transcripts from clinical parasite isolates from Kenya. Though diverse, the DBLα domain present in most genes in 3D7 can be readily amplified using a set of universal primers [29]. We demonstrate a high degree of underlying similarity between the distributions of *var* sequences in Kenya and *var* sequences present within the genome of a fully sequenced parasite isolate 3D7. Second, we show how specific sequence features can be used to classify the sequences into groups that allow the *var* expression patterns of different clinical parasite isolates to be compared directly.

## Results

A total of 1,746 var DBLα clones were successfully sequenced from 12 field isolates using the DBLαAF′ and DBLαBR primers (Figure 1A;. see Table S1 for patient information). Of these, 722 clones were sequenced from cDNA and 1,024 from genomic DNA (Table S1; see Dataset S1 for a complete list of the sequences). Overall, a total of 878 non-identical sequences were identified (see Text S1). The sequences were too diverse for direct comparisons between isolates, and there was virtually no overlap between sequences of different isolates. We therefore focused on features from these sequences that would provide more general information about the *var* genes to which they belong.

### DBLα Sequences Contain Semi-Conserved Features

Smith et al. [30] have previously noted from the analysis of *var* sequences from laboratory isolates that the different domains of *var* genes contain islands of homology that can be used to distinguish different classes of DBLα domains, called DBLα and DBLα1. Within the region we amplified, the positions that were most discriminatory between DBLα and DBLα1 domains corresponded with two cysteine residues [27]. We therefore analysed the amino acid composition of the amplified sequences from Kilifi, Kenya. For all the amino acids apart from cysteine, the frequencies were distributed around single modal values (data not shown). In contrast, the majority of DBLα sequences contained either two or four cysteine residues, with only a minority containing one, three, five, or six cysteines (Figure 1B; such sequences are hereafter referred to as cys2, cys4, and cysX types, respectively). This is entirely consistent with the 3D7 *var* genes. DBLα1 sequences are cys2 while most DBLα sequences are cys4. The functional importance of cysteine residues within the DBLα domain is supported by the observation that parasites from severe malaria cases from Brazil tend to express *var* genes containing DBLα domains with reduced numbers of cysteine residues [31].

Next, amino acid motifs occurring at four fixed positions within the sequenced regions were chosen. These will be referred to hereafter as positions of limited variability 1 to 4 (PoLV1–4; Figure 1C). Each PoLV was four amino acids in length and situated directly adjacent to conserved amino acid residues at the fringes of the previously defined islands of homology. Thus, each PoLV was located at identical relative positions within each DBLα sequence. The PoLV motifs and cysteine count were used as features to classify the DBLα sequences further.

### Sequences from Kilifi and from a Laboratory Isolate Contain Similar Distributions of Semi-Conserved Features

**Figure 2:**
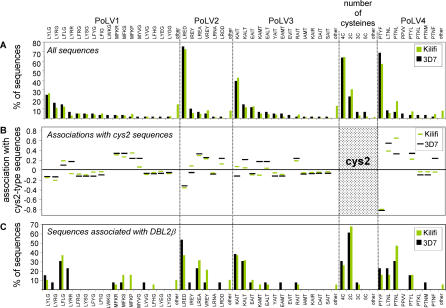
Conservation of Sequence Features between 3D7 and Kilifi Field Isolates (A) Distribution of sequence features within the 3D7 genome (black bars) and in Kilifi sequences (green bars). Sequence features not shared between Kenyan isolates and 3D7 are marked “other”. (B) Relationships between sequence features in sequences from Kilifi and 3D7: distribution of sequence features among cys2 sequences relative to those with non-cys2 sequences. The Cramer's *V* statistic (*y*-axis) indicates whether each of the listed sequence features was positively (*V* > 0) or negatively (*V* < 0) associated with cys2 sequences. Sequences from Kilifi are indicated with green bars; those from 3D7 are indicated by black bars. (C) The distribution of DBLα sequence features within *var* genes containing a DBL2β domain. PCR was performed on genomic DNA from two field isolates, 4161 and 4111 (see Figure 1A for amplification details). The percentage of distinct sequences containing each of the features listed is shown for Kilifi sequences (green bars) and is compared to the distribution of similar sequences from the 3D7 genome sequence (black bars).

The distribution of DBLα features between different *var* genes was examined in the full genome sequence of 3D7 [1]. In the entire 3D7 repertoire of 59 *var* genes there are 17 variants of PoLV1, six of PoLV2, 13 of PoLV3, and eight of PoLV4. We compared the DBLα features in 3D7 with those found among different Kilifi sequences. The majority of Kilifi sequences contained PoLV motifs that were found in the 3D7 genome (Figure 2A; Text S2). Furthermore, there was a close similarity between the distribution of PoLV motifs among *var* genes from the 3D7 genome and among the Kilifi sequences (Figure 2A). In both sets of sequences a similar hierarchy was evident in the frequency of variants of each sequence feature, with the same features being common and rare in each. The similarity between Kilifi and 3D7 sequences extended to the associations between different DBLα features. For example, Figure 2B shows the tendency of the different PoLV motifs to be associated with cys2 sequences. The same PoLV motifs tended to be associated with cys2 sequences among both 3D7 and Kilifi sequences, and the overall pattern of positive and negative associations was strikingly similar (Mantel-Haenszel test, *p* = 0.000002).

To test whether similarity between Kilifi and 3D7 sequences extended as far as the next downstream DBL region (see Figure 1A), genomic DNA from two field isolates, 4111 and 4161, was amplified with primers DBLαAF′ and BetaR located within the DBLα and DBL2β regions, respectively. Cloned PCR products were sequenced at both ends to determine the DBLα sequence at the 3′ end and confirm correct priming within DBL2β at the 5′ end. Figure 2C shows the distribution of DBLα features among distinct sequences. Among these clones there was a clear bias towards sequence features that are associated with DBL2β in the 3D7 genome, namely PoLV1_LFLG_, PoLV2_LREA_, PoLV4_PTNL_, and cys2. A similar conservation was evident upstream (see Text S3). Taken together, these observations suggest that despite being extremely diverse, DBLα sequences are built around a finite collection of building blocks whose relationships with one another follow underlying ground rules.

### Assignment of DBLα Sequences to Groups

**Figure 3:**
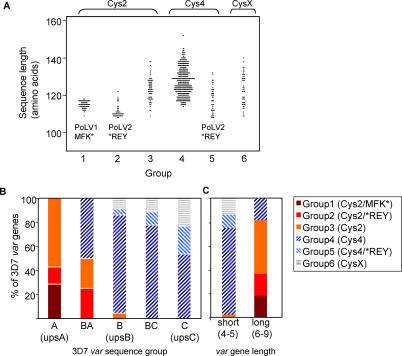
Sequence Groups (A) DBLα sequences were divided into six sequence groups: sequence groups 1–3 are those that contain two cysteine residues (cys2), and sequence groups 4 and 5 are sequences that contain four cysteine residues (cys4). Sequence group 6 includes sequences with one, three, five, or six cysteines (cysX). Sequence groups 2 and 5 contain PoLV2_*REY_. Sequence group 1 contains PoLV1_MFK*_. The length of each distinct DBLα sequence within each sequence group is indicated. (B and C) The distribution of 3D7 *var* genes in each DBLα sequence group among groups previously defined on the basis of coding and upstream non-coding regions of full-length *var* sequences [1,33] (B) and the overall length of the genes (C). Genes are classified as short if they have 4–5 domains and long if they have 6–9 domains (see Text S6).

To simplify comparisons between the different isolates and to summarize the profile of expression, we sought an algorithm to assign the sequences to groups. Though in 3D7 cys2-type DBLα sequences correspond very well with those that were previously classified as DBLα1 [27], we did not expect to identify additional discrete subgroups of sequence because of the high frequency of recombination between *var* genes [20,21,32]. However, inspection of the sequences suggested an approach to identifying subgroups. As shown in Figure 1B, cys2 DBLα sequences were significantly shorter than cys4 DBLα sequences ( Mann-Whitney *U* test, *p* < 0.0001). This is consistent with these forming distinct sequence groups. We considered the possibility that additional sequence features may exist that are independently associated with DBLα sequence length. Using logistic regression analysis, two such groups of sequence features were identified (see Materials and Methods and Text S4). These were PoLV1_MFK*_ and PoLV2_*REY_ (with the asterisk marking degenerate positions). PoLV2_*REY_ was associated with short sequences in both cys2 and cys4 sequences. PoLV1_MFK*_ was found exclusively in cys2 sequences and was independently associated with short sequences (Figure 3A). Among the cys2 sequences there was a complete absence of sequences that contained both PoLV1_MFK*_ and PoLV2_*REY_. This is a significant departure from a random distribution (Fisher's exact test, *p* < 0.001), suggesting that these features define subgroups of cys2 sequence.

We used this information to assign each sequence to one of six groups (Figure 3A). Since discrimination by number of cysteine residues corresponded well with the previous classification of DBLα regions from 3D7 [27], sequences were first divided into cys2, cys4, and cysX sequences. Cys2 sequences were then divided into those containing PoLV1_MFK*_ (group 1), those containing PoLV2_*REY_ (group 2), and those containing neither (group 3). Cys4 sequences were divided into those without PoLV2_*REY_ (group 4) and those with PoLV2_*REY_ (group 5). CysX sequences were placed in group 6. Thus, groups 1, 2, and 5 were strictly defined using two features, groups 3 and 4 were defined with one feature, and group 6 contained the remaining unusual sequences.

We tested this system of classification on full-length *var* gene sequences from the 3D7. The full-length *var* genes have previously been classified into five major groups (A to E) using both coding and upstream non-coding regions [1,28,33]. Figure 3B shows how DBLα sequences from 3D7, classified using our algorithm, are distributed between the five *var* gene groups. Figure 3C shows how the DBLα sequences are distributed between short and long *var* genes. There were striking differences in the distribution of DBLα sequences particularly comparing group A, B, and C *var* genes and long and short *var* genes (see Text S3).

**Figure 4:**
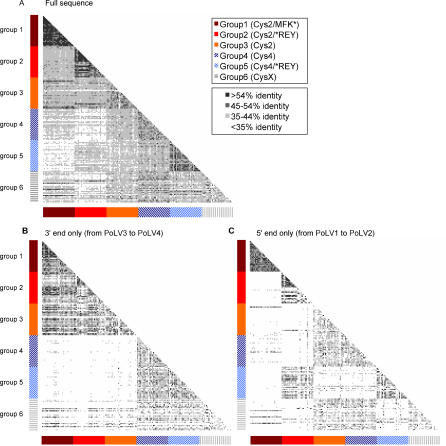
Global Comparisons of Sequences Falling in Six Sequence Groups Using ClustalW, pair-wise sequence identity comparisons were made between 30 randomly selected, distinct sequences from each sequence group. Pair-wise comparisons are expressed in an identity matrix, in which the percent identity between pairs of sequence is represented in different shades of gray. (A) Full-length sequence comparisons. (B) Sequence comparisons of the region from the 5′ end of PoLV3 to the 3′ end of PoLV4. (C) Sequence comparisons of the region from the 5′ end of PoLV1 to the 3′ end of PoLV2.

To determine the relationships between the six *var* groups, 30 randomly chosen sequences from each group were globally aligned using ClustalW analysis. A pairwise identity matrix was then constructed with the sequences sorted into their six groups (Figure 4A). It is clear from this comparison that, despite being defined using only a small amount of sequence information, groups 1, 2, and 5 form discrete sequence groups, since more sequence identity is shared between members of the same group than between groups. The distinction between groups 1 and 5 is particularly striking. Though groups 3 and 4 do not appear to form such discrete groups, the distinction between groups 2 and 4 was marked. Group 6 does not define a discrete sequence subset when analysed globally in this way, and may contain sequences derived from a variety of different recombination events.

Overall, this identity matrix suggested a complex web of relationships between the different sequence groups. Visual inspection of the sequences suggested that the similarity between groups 2 and 5 extended 5′ from PoLV2_*REY_. Therefore, to explore the relationships between these groups, we generated two further identity matrices, the first comparing the region from the 5′ end of PoLV3 to the 3′ end of PoLV4 (Figure 4B) and the second comparing the region from the 5′ end of PoLV1 to the 3′ end of PoLV2 (Figure 4C). These two comparisons gave strikingly different pictures of the interrelatedness of these groups. From Figure 3B it is clear that cys2 sequences (groups 1–3) are distinct from cys4 sequences (groups 4 and 5). However, Figure 3C shows that this distinction breaks down in the regions between PoLV1 and PoLV2. Thus, groups 2 and 5 and groups 3 and 4 share some identity within this region. From this analysis it is unclear whether these two related pairs of groups originated from ancestral hybrid sequences or whether recombination between cys2 and cys4 still occurs. What is clear is that group1 is distinct from cys4 sequences over the entire length of the sampled region.

### Sequence Groups Are Consistently Represented in Genomic DNA from Clinical Isolates but Are Differentially Expressed

**Figure 5:**
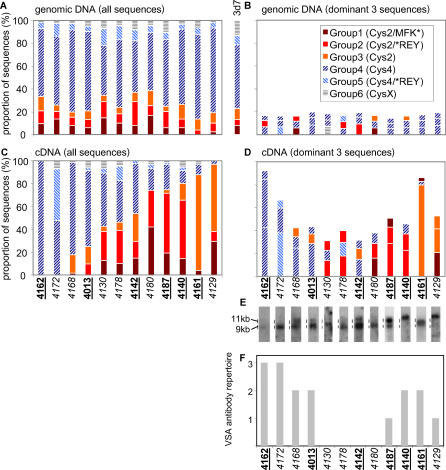
*var* Gene Expression Profiling (A–D) Each DBLα sequence was assigned to one of six sequence groups (Figure 3). The proportion of clones that fell into each of the six groups was calculated separately for genomic clones (A) and cDNA clones (C). (A) includes the distribution of sequences from the 3D7 genome (right). (B) and (D) show for each isolate the percent of genomic DNA (B) or cDNA clones (D) corresponding to the three most dominantly cloned genomic or cDNA sequences from that isolate. Isolates are ordered left to right according to the overall proportion of cys2 clones isolated from cDNA. Underlined ID numbers correspond to children with severe malaria. (E) Northern blots of total RNA from each of the parasite isolates. Blots were hybridized to a generic *var* exon 2 probe, *var*c, corresponding to a conserved region within all *var* genes. The position of *var* genes expressed by the laboratory parasite line Palo Alto is indicated by lines to the left of each lane. These are approximately 9 kb and 11 kb in length. (F) The VSA antibody repertoire carried at the time of acute disease by each patient. The *y*-axis shows the number of a panel of six parasite isolates that were recognised by the acute plasma from each child.

Using the above system of classification each of 12 clinical parasite isolates were compared (Figure 5). Figure 5A and 5C divide all 1,746 DBLα sequences by (1) whether they were cloned from genomic DNA (Figure 5A) or cDNA (Figure 5C), (2) the parasite isolate from which they were isolated, and (3) the group to which they were assigned. The cloning frequencies of genomic sequences from each group were fairly constant between parasite isolates and close to those expected from the distribution of *var* genes in the 3D7 genome (Figure 5A). This suggests that the number of sequences from each group was relatively constant between different parasite genomes. In contrast, there was considerable variation in the cloning frequency of cDNA-derived sequences (Figures 5C and S1). To highlight this, parasite isolates were sorted left to right according to increasing cloning frequency of cys2 cDNA sequences. Between the 12 isolates there was a significant correlation between the expression of group 1 and group 2 (*r*_s_ = 0.67, *p* = 0.02), suggesting that they may be under similar expression control.

Based on their distribution within the 3D7 genome (see Figure 3C), we expected cys2 sequences to be associated with long *var* genes [1]. To confirm this, Northern blots of total RNA from each parasite isolate were hybridized to a generic *var*-specific probe from the relatively conserved 5′ exon 2 region. There was a good correspondence between the size of the bands (Figure 5E) and the dominant cDNA sequences from each isolate (Figure 5D). In five of seven samples that had a dominant band less than or equal to 9 kb in length, the dominant sequence was cys4 type. In five of five samples that had a dominant band greater than 9 kb, the dominant sequence was cys2 type (Fisher exact test, two-tailed, *p* = 0.03).

### The Parasite Rosetting Phenotype Is Associated with Expression of Group 2 Sequence

**Figure 6:**
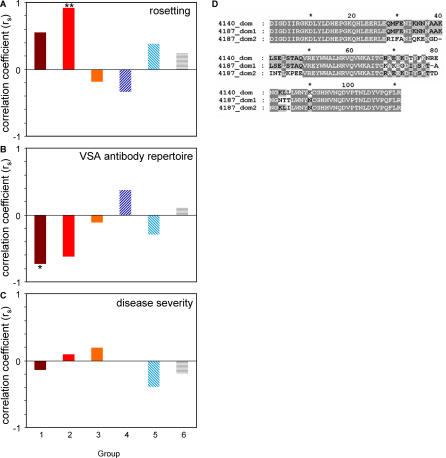
Relationships between the Expression of Each DBLα Sequence Group and Markers of the Host–Parasite Relationship In each graph the Spearman's rank correlation coefficient (*r*_s_) is shown for each sequence group. Significance without Bonferroni correction is indicated as follows: *, *p* < 0.05; **, *p* < 0.001. (A) Correlation between expression of each sequence group and parasite rosetting (percent of infected erythrocytes forming rosettes). (B) Correlation between expression of each sequence group and host VSA antibody repertoire (the number of a panel of six isolates recognised by the patient plasma). (C) Correlation between expression of each sequence group and severe malaria. (D) Alignments of sequences associated with parasite rosetting. Similar sequences were found to be dominant in two isolates (4140 and 4187) with the highest rosetting frequency. In isolate 4187, the two most dominant sequences (4187_dom1 and 4187_dom2) were highly similar. All three sequences shown have the same sequence signature, “sig2”: LYLD-VERY-KAIT-2-PTNL.

As a test of the validity of our DBLα sampling and grouping strategy, we tested whether the parasite rosetting phenotype is associated with expression of specific DBLα sequence groups. Since this phenotype is mediated by the DBLα domain of PfEMP1 [12,13], we expected that specific sequence features associated with rosetting may be associated with the sequence features used to define our groups. In support of this, a striking positive association was observed between group 2 expression and the percentage of infected erythrocytes that formed rosettes (*r*_s_ = 0.92, *p* < 0.001, corrected for six comparisons (Bonferroni); Figures 6, S2C, and S2D). Furthermore, the two parasite isolates with the highest rosetting rates expressed dominant group 2 sequences with the same combination of sequence features. (i.e., the sequence “signature”; see Materials and Methods, Text S5, and Figure S3 for more details; this particular sequence signature was called “sig2” in Figure S3). These highly similar sequences are shown in Figure 6D.

### *var* Gene Expression in the Infecting Parasite Population Reflects the Host VSA Antibody Response at the Time of Disease

Previous studies predicted that as children build up a repertoire of anti-VSA antibodies, the proportion of VSAs that can be expressed by the infecting parasite population is diminished [16]. More recently, mathematical modelling has suggested that sequential expression of single VSAs can be sustained by the anti-VSA antibodies [34]. Between parasite isolates in this study, there was considerable variation in the extent to which the cDNA sequences were dominated by a small number of sequences. Figure 5D shows the extent to which the most dominant sequences from each parasite isolate accounted for the entire collection of clones from that isolate among cDNA sequences (i.e., the homogeneity of the collection of sequences; see Materials and Methods). From Figure 5D it is clear that among the cDNA clones, dominant sequences were identified from each of groups 1–5 with no striking association between any particular group and the disease severity of the infected child (see also Figures 6C, S1, S2A, and S2B).

If the expressed *var* genes correspond with VSAs expressed on the infected erythrocyte, then, following from previous studies, we would expect to observe a positive association between the homogeneity of the *var* message and the repertoire of VSA antibodies carried by each child at the time of disease (see Figure 5F). In support of this, there was evidence for such an association among the cDNA sequences (*r*_s_ = 0.81, p = 0.002; Figure 5D) but not among the gDNA sequences (*r*_s_ = −0.37, p = 0.23; Figure 5B). Previous serological studies have further led to the suggestion that a subset of relatively conserved VSAs is under particularly high immune selection [17,18]. To test whether any of the DBLα groups show evidence of being under high immune selection relative to the other groups, we tested for a negative association between the relative expression of each of the groups and the VSA antibody repertoire of the infected child. Evidence for such an association was found for sequence group 1 (*r*_s_ = −0.68, *p* = 0.015, *p* = 0.088 after Bonferroni correction for six comparisons; see Figures 6B, S2E, and S2F). Though this association clearly needs to be confirmed in larger studies, the fact that group1 sequences were relatively well conserved between isolates agrees with predictions from the previous serological data.

## Discussion

Despite years of research very little is known about how the host–parasite relationship changes as naturally acquired anti-malarial immunity develops. More specifically, we lack molecular tools for measuring changes in the parasite as it adapts to the development of clinical immunity in vivo. Such tools could provide a powerful means of dissecting the protective components of host response, a first step in the identification of new vaccine candidates. A main requirement for such tools is that they can be used in field-based studies. Here we have assessed a simple approach using large-scale sequencing of short stretches of sequence from DBLα, a region that, though highly diverse, is present in the majority of *var* genes.

Expectations that such an approach could generate data that would reflect the host–parasite relationship at the time of disease have until recently been low. This has been due to uncertainty about whether the high recombination rate between *var* genes and their extreme diversity would allow meaningful comparisons between isolates [20,21,32]. The 3D7 genome sequence [1] has provided more encouraging information. The location of *var* genes in both internal and telomeric locations and their mixed direction of transcription set up the conditions for genetic structuring. This is supported by the existence within the 3D7 genome of two groups of *var* genes encoding PfEMP1 with different functional properties [27]. The two groups of genes carry different DBLα sequences defined as DBLα and DBLα1 [27]. These observations opened up the possibility of obtaining functionally relevant data from field isolates using only limited sequence data. However, it was uncertain how useful these definitions would be to field studies.

Here we analysed a large number of different sequences from the DBLα region of *var* genes from Kilifi, Kenya, focusing on a limited number of semi-conserved sequence features. The data strongly support the existence of an underlying order that extends from the single genome to the parasite population as a whole. This enabled us to use the 3D7 genome as a basic reference for interpretation of the field data. Overall, the similarity between Kilifi and 3D7 sequences was extensive, in terms of (1) the range of sequence features observed and their similar frequency distribution, (2) their relationships with each other within the sequence, and (3) their relationship to features outside the sequence region that was sampled. Most notably, within the region we have sequenced the main defining feature of DBLα1 sequences in 3D7 is the existence of two cysteine residues (cys2) rather than the normal four cysteines (cys4). In the field isolates, apart from a small minority, the sequences could be classified as either cys2 or cys4. These observations together help clarify an interesting earlier observation from Brazil, where DBLα sequences containing reduced numbers of cysteines (corresponding to our cys2 sequences) tended to be expressed in children with severe malaria [31].

Comparison of the lengths of the DBLα sequences revealed that cys2 types were significantly shorter than cys4 types. Other sequence features independently associated with sequence length were subsequently identified and used to place the sequences in groups, providing a simple means of classifying the sequences. The practical usefulness of these groupings is supported by the striking association between sequence group 2 expression and parasite rosetting. The rosetting phenotype is a well established virulence phenotype mediated by binding of a subset of DBLα domains to complement receptor 1 (CR1) on erythrocytes and has been found in several previous studies to be associated with severe malaria [35,36]. Surprisingly, these sequences were not related to previously identified rosetting *var* genes such as R29 [12] or FCR3S1.2*var*1 [13], which fall in groups 1 and 4, respectively, suggesting that they may represent a novel class of rosetting *var* genes.

In most of the isolates there were clear dominant cDNA sequences, and the dominance of particular sequences in different infections was consistent with previous studies of *var* gene expression from field isolates [37,38]. This challenges previous ideas based on studies of *var* gene expression in laboratory isolates. Previous studies suggested that all *var* genes may be switched on in the immature ring stages, but only one is expressed in mature stages. These data suggested a post-transcriptional level of control that would prevent meaningful data being obtained from uncultured parasite isolates [39–41]. Though Kaestli et al. [38] took the precaution to pre-select for full-length transcripts to remove the possibility of amplifying incomplete transcripts, neither Peters et al. [37] nor ourselves performed this step, suggesting that background transcription may not be a cause for concern in the interpretation of field studies.

The primary aim of sampling *var* gene sequences from clinical isolates was to use the information to track changes in *var* gene expression associated with the development of naturally acquired immunity to malaria. It is important for such studies to be carried out over a long period of time and in different geographical locations. However, as a first step, it was encouraging to find that the repertoire of VSA antibodies carried by a child at the time of disease correlated with both the tendency of the cDNA sequences to be dominated by a small number of sequences, and their bias away from a small group of relatively conserved cys2 sequences (group 1). Both these observations fit in well with previous serological and theoretical studies that suggest that theVSA antibody response both supports sequential expression of single VSAs [34] and selects against those that are most conserved. Previous serological studies have led to the suggestion that a restricted subset of commonly recognised PfEMP1 molecules are associated with both low host immunity and severe malaria [17,18,23]. In an attempt to select for the expression of such molecules in vitro, Jensen et al. [42] selected the 3D7 parasite line on IgG from malaria-exposed children. Several *var* genes appeared to be specifically selected by these naturally acquired antibodies. The DBLα tag regions of the majority of these were found to be cys2 sequences, though not specifically from group 1. A key question for future research is why certain *var* genes would be maintained in the genome if they are particularly sensitive to immune selection. If such genes have specific functional properties, it would be important to examine these in detail to assess their potential usefulness as vaccine candidates.

However, in the present study there was no clear evidence for any particular sequence group being associated with severe malaria. As noted above, Kirchgatter et al. [31] previously observed that children with severe malaria tend to express DBLα sequences with cys2 sequences, and Bian et al. [43] have observed that parasites causing severe malaria tend to express long PfEMP1 molecules [43]. In the 3D7 genome both these are characteristics shared by *var* genes that lie downstream of upsA control elements (see Figure 1A). These observations together with those of Jensen et al. [42] may suggest a specific role for upsA *var* genes in severe malaria. In the present study, the clear bias in parasites from two severe cases away from expression of cys2 DBLα sequences suggests that some caution is needed in regard to this interpretation. However, the strong association of a subgroup of cys2 sequences (group 2) with rosetting and the observation that parasites from two of the six severe cases expressed very similar group 2 dominant sequences are consistent with the idea that some children with severe malaria express a restricted subset of cys2 *var* genes. More samples are clearly needed to confirm this observation.

In future studies with larger numbers of parasite isolates it will be interesting to explore DBLα expression patterns in relation to other aspects of the host–parasite interaction, such as the number of parasite genotypes present, host endothelial cell binding phenotype, and various components of the host immune response. Though initially it would be important to carry out these studies using DNA sequence data, the fact that sequence groups can be defined using short sequence motifs suggests that approaches based on microarray and real-time PCR analysis could be developed to distinguish between the expression of different groups of *var* sequences. In addition, the close relationship between PCR product length and the sequence group of the products raises the possibility that inexpensive approaches to *var* expression typing might be developed using PCR product length data.

In conclusion, we have shown that *var* genes from both field and laboratory isolates can be classified into biologically meaningful subsets based on small blocks of semi-conserved sequence. Further sequencing of *var* genes from a much larger number of parasites derived from patients that have been rigorously categorised with respect to clinical presentation and parasite phenotype is clearly necessary. By focusing attention on subgroups of *var* genes that are associated with parasite virulence and host immune status, such studies may provide further information with implications for malaria intervention.

## Materials and Methods

### Study site.

The study was carried out at Kilifi District Hospital, situated 50 km north of Mombasa on the coast of Kenya. The hospital has a high-dependency ward to treat children with severe life-threatening malaria, a paediatric ward to treat children with moderate malaria, and an outpatient department to treat children with mild malaria.

### Sample collection.

Children were recruited if they had a primary diagnosis of malaria and parasitaemia ≥ one trophozoite per 100 uninfected erythrocytes [17]. Isolates were collected and white blood cells removed as described previously [22]. For each isolate a sample of acute plasma was stored at −20 °C. Parasites were collected from children attending hospital between July 1998 and February 1999 and have been described previously [17,44]. Twelve patients were selected for the study: six with mild disease and six with severe disease.

### Serotyping of plasma.

Plasma from each of the 12 patients was tested by agglutination assay against six parasite isolates from blood group O individuals (ID numbers 4513, 4518, 1759, 4542, 4508, and 4528) who came to hospital with malaria either between January and August 2000, or, in the case of 1759, in December 1995 [44]. The VSA antibody repertoire carried by these plasma samples was defined as the number of the six target isolates that were agglutinated.

### Agglutination assays.

Parasites were cultured until they were middle to late pigmented trophozoites, as described previously [22]. Assays were performed in microtitre plates (Falcon, Becton-Dickinson, Palo Alto, California, United States) at 4% haematocrit in RPMI at a parasitaemia of 1–2 trophozoites per 100 uninfected erythrocytes in a 12.5-μl total assay volume in the presence of 2.5 μl of plasma. Cells were rotated for 1 h as described previously [22]. Assays were scored using the dry agglutinate method as described previously [17].

### Rosetting assay.

Cells (0.5 μl) were resuspended in 9.5 μl of RPMI containing 5 μg/ml acridine orange. Following the addition of 2.5 μl of non-immune European serum, cells were rotated for 30 min on a vertical rotator and the entire reaction volume pipetted onto a glass slide, covered with a coverslip, and observed under a fluorescence microscope (Nikon, Tokyo, Japan). Rosetting was scored as the percentage of 100 mature trophozoites that adhered to at least two uninfected erythrocytes.

### Characterization of DBLα sequences.

Pellets (100 μm) of packed infected erythrocytes were collected and, following lymphocyte and phagocyte depletion, were stored in Trizol (Invitrogen, Paisley, United Kingdom) at −30 °C. RNA was prepared as described previously [45]. To amplify *var* from RNA, the RNA was first treated with DNAse I (DNAse Free, Ambion, Cambridge, United Kingdom) according to the manufacturer's instructions. The DNAse was removed using Ambion DNase inactivation reagent. RNA (2 μl) was reverse transcribed using reverse transcriptase (Invitrogen SuperscriptII). For each isolate a negative control reaction was performed in the absence of reverse transcriptase to ensure that all contaminating DNA had been removed by DNAseI pre-treatment. Sufficient DNA was present in the untreated RNA sample for PCR amplification of genomic DNA. Suspended sample (1 μl) was diluted in 10 μl of water, and 1 μl was amplified directly by PCR. DBLα sequences were amplified with the following primers: DBLαAF′, GCACG(A/C)AGTTT(C*/T)GC, and DBLαBR, GCCCATTC(G/C)TCGAACCA, modified from [29] (Figure 1A). The nucleotide marked with an asterisk indicates a modification from the originally described primer DBLαAF. This change was introduced to broaden the range of sequences that can be amplified. PCR amplifications between DBL1α and DBL2β were performed using the following primers: DBLαAF′, see above, and BetaR, GA/CCCAC/TTCIGC/TCATCCA. The following conditions were used. For isolation of DBLα sequences, 35 cycles of PCR were performed in 25 μl using an annealing temperature of 42 °C and a 30-s extension time at 65 °C in the presence of 0.2 U of Amplitaq polymerase (Applied Biosystems, Foster City, California, United States) and 3 mM MgCl_2_ to give a product of 400 bp. Amplification between DBLα and DBL2β was performed in the presence of BioXact polymerase (Bioline, London, United Kingdom) in the presence of 3 mM MgCl_2_ with an annealing temperature of 50 °C and extension time of 2 min at 65 °C to give a product length of approximately 2.3 kb. Following PCR, DBLα sequences were purified using Sephacryl (Amersham Biosciences, Amersham, United Kingdom). Products obtained by amplification between DBLαAF′ and BetaR were size selected on an ethidium-bromide-stained agarose gel and purified using a Qiagen (Valencia, California, United States) gel extraction kit. DNA was ligated into either TA vector or TOPO vector (Invitrogen) and used to transform TOP10 cells. From each clinical isolate we aimed to sequence approximately 100 genomic DNA and 50 cDNA DBLα clones. Sequencing was carried out using M13 reverse and T7 primers (3 pmol) with BigDye Terminator v3.1 cycle sequencing kit (Applied Biosystems). Samples were run on Applied Biosystems 3700 or 3730 sequencing machines.

### Amplification of DNA upstream of DBLα.

The following primers were used to test the relationship between (1) DBLα sequence features PoLV1_MFKR_ (amino acid sequence motif MFKR at PoLV1; see Figure 1C) or PoLV4_PTYF_ and (2) upstream sequences upsA or upsB (see Figure 1A). Reverse primers MK3, TCATTACGTTTAAACATATC (specific to PoLV1_MFKR_), and PTYF3′, ACGTAGTCAAAATATGTGG (specific to PoLV3_PTYF_); forward primers upsA750, AACATKGTTCTATTTTCTC, and upsB, TTGCCTCTDTTGTTATCTC, specific to upsA and upsB, respectively. All reactions were performed in the presence of 3 mM MgCl_2_ using an annealing temperature of 47 °C, 35 cycles, and an extension time of 1 min at 65 °C with Amplitaq polymerase in the presence of Taqstart reagent (Clontech, Becton-Dickinson). See also Text S3.

### Selection of sequences for analysis.

DNA subclones were selected for analysis if at least one of the pair of sequence reads contained a single open reading frame and began and ended within previously identified “homology blocks” of DBLα [30]. From the pair of sequence reads from each clone the best quality single read was chosen for analysis. Sequences selected for analysis were all open reading frames beginning at the position of the 5′ consensus motif DIGDI within homology block D and ending at the position of the 3′ consensus motif PQYLR within homology block H. Five different sequences (eight clones in total) were excluded from the analysis because they were from non-alpha DBL domains.

### Extraction of sequence features.

Translated sequences were aligned in batches using ClustalW analysis (http://www.ebi.ac.uk/clustalw/) using the default settings (Gonnet250 matrix, gap opening penalty = 10.0, gap extension penalty = 0.2, gap closing penalty = −1, gap separation penalty = 4). Sequence features listed in Figure 1C were extracted from the sequence using GeneDoc software (http://www.psc.edu/biomed/genedoc/) and exported into Microsoft (Seattle, Washington, United States) Excel and Stata version 6.0 (StataCorp, College Station, Texas, United States) for further analysis.

### Sequence analysis.

To test the association between sequence features within DBLα sequences, the Cramer's *V* statistic was used in Stata. This is a representation of χ^2^, but is bounded between −1 and +1. To identify sequence features that were independently associated with DBLα sequence length, the following strategy was used. (1) The association between each PoLV motif and sequence length was determined using the Mann Whitney *U* test. (2) PoLV motifs with a highly significant negative association with sequence length (*p* < 0.0001) were identified. (3) These sequence features were grouped allowing one degenerate position. (4) Logistic regression was used to screen each DBLα sequence feature or group of features simultaneously for those that were independently associated with sequence length. See Text S4 for more details.

### Sequence signatures.

Because of the high overall sequence diversity, very few of the sequences had absolute matches between isolates. To make more general comparisons between different sequences and to identify common and rare sequence types within each group, DBLα sequence features were used to reduce each sequence to a “signature” of standard length. The signature consisted of the string of amino acids at each of the PoLVs together with the cysteine count (see Figure 1C for examples). Sequence signatures are discussed further in Text S5 and Figure S3.

### Definition of “distinct” sequences.

Several of the analyses described here, in particular the identification of sequence groups, were performed on collections of “distinct” sequences. A robust definition of “distinctness” was required to help minimise repeated sampling of very similar sequences arising from PCR and sequencing errors. For this definition, two sequences were considered distinct if they had either (1) non-identical signatures or (2) different amino acid length.

### Homogeneity of cDNA expression.

Homogeneity of cDNA expression was defined for each isolate as the total number of cDNA clones containing the dominant two sequences from that isolate, expressed as a percentage of all cDNA clones sequenced from that isolate.

### Northern blot analysis.

For comparison of full-length ring-stage *var* RNA transcripts, Northern blots were prepared and hybridized with a generic *var* exon 2 (see Figure 1A) probe as previously described [45]. A sample of laboratory isolate Palo Alto RNA was included, to allow size comparison between samples run on different gels. The largest commercially available markers go up to 9.5 kb, whereas the Palo Alto sample has major *var* transcripts at approximately 9 kb and 11 kb. Exposures to autoradiography film ranged from 1 to 4 d.

### Generation of sequence identity matrices.

Distinct sequences from each required category were picked at random using the RAND function in Microsoft Excel and subjected to ClustalW analysis as described above. Identity matrices were generated in the form of statistics reports using GeneDoc software and the report file saved with an *.xls extension. Files were opened in Microsoft Excel and conditional formatting was used to shade the matrix as follows: 80% gray (55%–100% identity), 50% gray (45%–54% identity), 25% gray (35%–44% identity), and white (< 35% identity).

## Supporting Information

Dataset S1 The 1,746 Translated DNA Sequences Used in the Study (248 KB TXT)

Figure S1 *var* Expression Profiling Pie charts are used to show the number of distinct sequences of each group cloned from each parasite isolate. The size of each slice is proportional to the number of clones of that sequence identified.
(19 KB PDF)

Figure S2 Global Analysis of Expressed DBLα Sequences between Subsets of Parasites To obtain a global picture of the expression of different groups of parasites and to test the usefulness of our sequence groupings we randomly selected 26 cDNA clones from each parasite isolate and constructed identity matrices of pair-wise comparisons of the sequences. Multiple sequence comparisons of all 312 sequences were first performed using ClustalW. The twelve isolates were then split into various groups of six, and pairs of identity matrices were constructed from the selected sequences as described in Materials and Methods: mild and severe cases (A and B), low and high rosetting (C and D), and VSA antibody positive and negative (E and F). The expression patterns further illustrate the associations described in the text and reveal subtle characteristics of expression patterns that need to be explored in future studies with larger samples of parasites. The most notable is the apparent emergence of large clusters of similar sequences in both rosetting parasites and those from antibody-negative children. The fact that this is not apparent in children with severe malaria may reflect heterogeneity in the *var* genes compatible with causing severe malaria. However, this can be tested only by comparisons using matrices generated from much larger pools of sequences.
(22 KB PDF)

Figure S3 Significant Sequence Signatures from Kilifi and Elsewhere Forty-seven Kilifi sequence signatures are shown of the total 393 isolated, in addition to two signatures from previously identified *var* genes associated with rosetting that were not found among Kilifi sequences. Kilifi sequences were considered significant based on four criteria: (1) being among the dominant cDNA sequences represented in the cDNA from each isolate (dark green boxes); (2) being sequence signatures that were isolated from more than five isolates (including 3D7); (3) being sequence signatures of full-length sequences that were identical in two or more isolates (highlighted with a white X); or (4) being sequence signatures shared with previously identified *var* genes of note. Only sequences cloned from cDNA and representing greater than 20% of all the cDNA clones from that isolate are shown as dark green squares. Expressed signatures that were not the most dominant sequence or were present in less than 20% of the sequences from each isolate are represented as light green boxes. For dominant and second most dominant sequence signatures from each isolate, the percentage of cDNA sequences containing that signature is indicated. Signatures only identified in genomic DNA from a given isolate are indicated as light gray boxes. The sequence signatures are divided into sequence groups 1–6 and sorted, with the sequence signatures that were most frequently shared between isolates at the top of each group. Within the sequence signatures listed on the left, individual sequence features that were not found in the 3D7 genome are highlighted with brackets. Sequence features that were the most frequently represented within all the clones are highlighted in bold. Those that were most frequently represented within cys2 sequences are written in blue. The PoLV1_MFK*_ features are highlighted in dark red, PoLV2_*REY_ features are highlighted in light red. Previously described *var* genes that contain the sequence features listed here are indicated on the right: AFBR41 in the 3D7 genome was found to be dominantly expressed in a vaccinated volunteer [37]. 3D7chr5*var* and FCR3*var*CSA are collectively known as *var*1. *var*1-like genes isolated so far tend to have either 3D7chr5*var*-like or FCR3*var*CSA-like sequence signatures [46–49]. The tendency of the dd2*var*1 gene to be conserved between isolates has been noted previously (S. Kyes, unpublished data). R29, FCR3S1.2-*var*1, and A4-AFBR19 are associated with parasite rosetting [12,13,50]. For more on sig1 and sig2, see Text S5.
(14 KB PDF)

Table S1 Parasite Isolates and Patients Used in This Study (57 KB DOC)

Table S2 Mann Whitney *U* Test Analysis of Associations between Sequence Features and DBLα Sequence Length (66 KB DOC)

Table S3 Logistic Regression Analysis of Associations between Sequence Features and DBLα Tag Sequence Length (29 KB DOC)

Text S1 PCR and Sequencing Errors (19 KB DOC)

Text S2 Comparison of PoLV between Kilifi Sequences and Isolate 3D7 (26 KB DOC)

Text S3 The Relationship between DBLα and ups (20 KB DOC)

Text S4 Screening for Sequence Motifs Associated with DBLα Sequence Length (21 KB DOC)

Text S5 Sub-Classification of DBLα Sequences by Their Sequence Signatures (21 KB DOC)

Text S6 *var*1 Genes (23 KB DOC)

### Accession Numbers

The EMBL Nucleotide Sequence Database (http://www.ebi.ac.uk/embl) accession numbers discussed in this paper are cDNA (AM114937-AM115658) and gDNA (AM115696-AM116719).

## Abbreviations

CIDR: cysteine-rich interdomain region
DBL: Duffy-binding-like
PfEMP1: *Plasmodium falciparum* erythrocyte membrane protein 1
PoLV: position of limited variation
VSA: variant surface antigen
