# Supplementary material for: Plasmodium falciparum Variant Surface Antigen Expression Patterns during Malaria
Source: PLoS Pathog. 2005 Nov 18;1(3):e26. doi: 10.1371/journal.ppat.0010026 (PMC1287908; doi:10.1371/journal.ppat.0010026)
Supplement: Supplementary file 1 [file ppat.0010026.sg001.pdf]

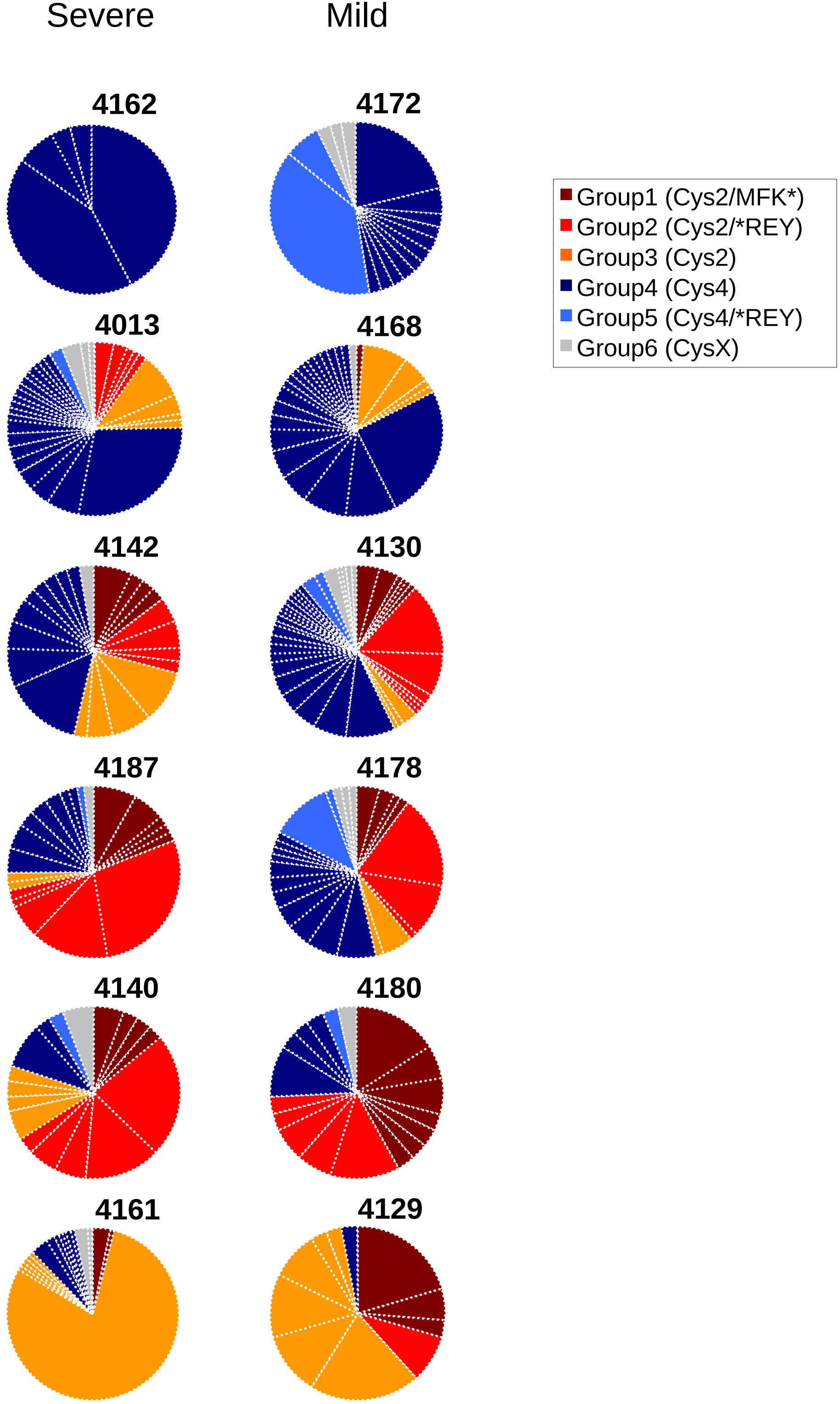

Severe
Mild
4162
4172
4013
4168
4142
4130
4187
4178
4140
4180
4161
4129
Group1 (Cys2/MFK*)
Group2 (Cys2/*REY)
Group3 (Cys2)
Group4 (Cys4)
Group5 (Cys4/*REY)
Group6 (CysX)
