# Supplementary material for: Plasmodium falciparum Variant Surface Antigen Expression Patterns during Malaria
Source: PLoS Pathog. 2005 Nov 18;1(3):e26. doi: 10.1371/journal.ppat.0010026 (PMC1287908; doi:10.1371/journal.ppat.0010026)
Supplement: Supplementary file 2 [file ppat.0010026.sg002.pdf]

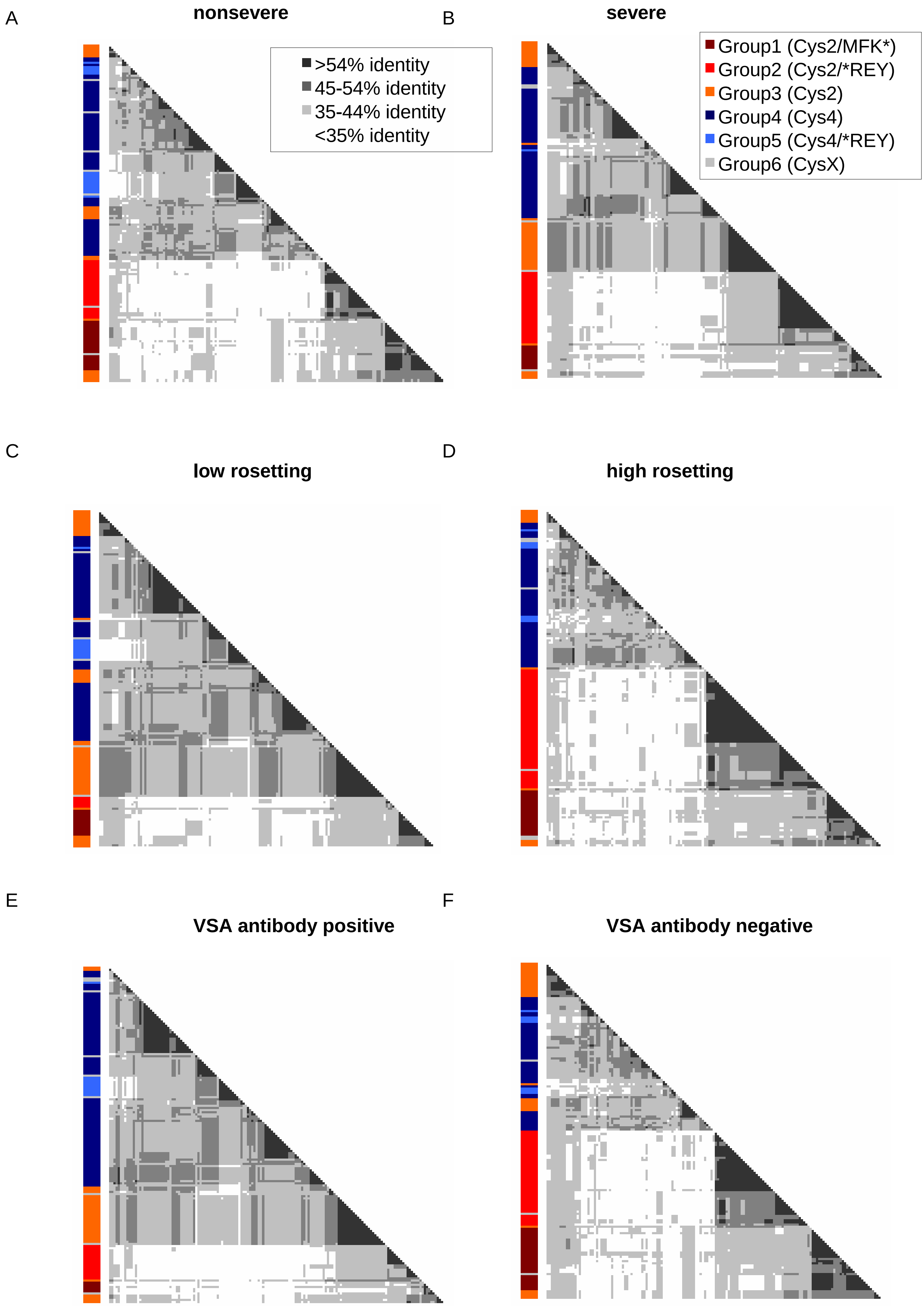
A
nonsevere
>54% identity
45-54% identity
35-44% identity
<35% identity
B
severe
Group1 (Cys2/MFK*)
Group2 (Cys2/*REY)
Group3 (Cys2)
Group4 (Cys4)
Group5 (Cys4/*REY)
Group6 (CysX)
C
low rosetting
D
high rosetting
E
VSA antibody positive
F
VSA antibody negative
