# Supplementary material for: Plasmodium falciparum Variant Surface Antigen Expression Patterns during Malaria
Source: PLoS Pathog. 2005 Nov 18;1(3):e26. doi: 10.1371/journal.ppat.0010026 (PMC1287908; doi:10.1371/journal.ppat.0010026)
Supplement: Supplementary file 3 [file ppat.0010026.sg003.pdf]

	3d7	4162	4013	4142	4187	4140	4161	4172	4168	4130	4178	4180	4129	
MFKS-LREA-KAIT-1-LTNL			x				x							
MFKP-LRED-KAIT-1-PTNL														A4-AFBR19
LYRR-VREY-KAIT-4-PTYF											12			
LYRG-VREY-EAIT-4-PTYF								38						
LYLG-LRED-KAIT-4-PTYF										9				sig1/dd2var1/A4-AFBR14
LFLG-LRED-KAIT-4-PTYF														
LYLG-LRED-KALT-4-PTYF				15	x	x				x				
LYLG-LRED-EAIT-4-PTYF														
LYRR-LRED-KAIT-4-PTYF														
LYRG-LRED-EAIT-4-PTYF														
LYSG-LRED-EAIT-4-PTYF									x			x		
LYLG-LRED-YAIT-4-PTYF														
LFIG-LRED-KAIT-4-PTYF														
LYSG-LRED-KAIT-4-PTYF														
LYRG-LRED-KAIT-4-PTYF														
LYLG-LRED-EALT-4-PTYF														
LFLG-LRED-EAIT-4-PTYF														
LFYG-LRED-KAIT-4-PTYF										9				
LFYG-LRKD-KALT-4-PTYF		x				x			x					
LFLG-(LRND)-(NAIT)-4-(LTNF)		x				x			x				x	
LYRG-LRNA-KAIT-4-PTYF		x				x				x				
LYRG-LRED-YAIT-4-PTYF		42												3D7-AFBR41
LYLG-LRED-KAIT-4-PTNF			28											
LYRG-LRED-RAIT-4-PTYF								21						
(LYFG)-LRED-KAIT-4-PTYF		42												
LFYG-LRED-EALT-4-PTYF								x	x					
LYSG-LRED-EALT-4-PTYF									10					
(LYIG)-LRED-KAMT-4-PTYL			x					x						
(MYVV)-LRED-EAIT-4-PTYF									25					
LYLG-LRED-GAMT-4-PTYF														FCR3S1.2-var1
LFYG-LRED-KAIT-2-PTNL													21	
LFIG-LRED-KAIT-2-PTNL			x						x					
LYLG-(LREH)-(QAIT)-2-PTNL		x						x						
LYLG-LRED-KAIT-2-PTYL							80							
LFLG-LREA-KAIT-2-PTNL			9											
LFLG-VREY-EALT-2-LTNL				x		x							x	3D7 chr5 var
(LYLD)-VREY-KAIT-2-PTNL					29	23								sig2
LFLG-VREY-KAIT-2-PTNL					14					15				
LFLG-VREY-(NAIT)-2-PTNL		x				x								
LFLG-IREY-EALT-2-PTNL											28			
LFLG-(LREY)-EALT-2-PTNL		x			x									
(MFLG)-VREY-KAIT-2-PTNL						14								
MFKP-LREA-EAIT-2-PTNL					x						x			FCR3varCSA
MFKP-LREA-KAIT-2-PTNL								x				x		
MFKP-LRED-RAIT-2-PTNL												16		
MFKS-LRED-RAIT-2-PTNL				x				x						
MFKS-LREH-EAIT-2-PTYL													21	
MFKP-LRED-KAIT-2-PTNL														R29
