# Supplementary material for: Plasmodium falciparum Variant Surface Antigen Expression Patterns during Malaria
Source: PLoS Pathog. 2005 Nov 18;1(3):e26. doi: 10.1371/journal.ppat.0010026 (PMC1287908; doi:10.1371/journal.ppat.0010026)
Supplement: Supplementary file 4 [file ppat.0010026.sd002.doc]

S1) 878 is an over-estimate of the actual number of distinct sequences since it does not take into account PCR and sequencing errors. When analyses of distinct sequences was performed a conservative definition of sequence uniqueness was required. For this, sequences were defined as different if they had a different combination of sequence features (sequence signature, see Materials and Methods) or different number of amino acids. Using this definition 611 different sequences were identified.
