# Supplementary material for: Plasmodium falciparum Variant Surface Antigen Expression Patterns during Malaria
Source: PLoS Pathog. 2005 Nov 18;1(3):e26. doi: 10.1371/journal.ppat.0010026 (PMC1287908; doi:10.1371/journal.ppat.0010026)
Supplement: Supplementary file 5 [file ppat.0010026.sd003.doc]

S2) Among the Kilifi sequences there were 55 variants of PoLV1, 25 of PoLV2, 39 of PoLV3 and 33 of PoLV4. 83% of distinct Kilifi sequences contained PoLV1 motifs found in 3D7, 92% contained 3D7 PoLV2 motifs, 86% and 91% contained 3D7 PoLV3 and PoLV4 motifs respectively. Only 3 PoLV motifs found in the 3D7 genome were absent from the Kilifi sequences (Figure 2A). Two of these (PoLV1_LWKG_, denoting the amino acid sequence LWKG at PoLV1, and PoLV3_EVIT_) are unique to a particular class of *var* gene called “var2CSA” [1]. The other (PoLV4_PPVV_) is found only in the “type 3” *var* genes [2]. In 3D7 neither genes have the DBLαAF and DBLαBR priming sites that would allow them to be amplified.

**References**

1. Salanti A, Staalsoe T, Lavstsen T, Jensen AT, Sowa MP, et al. (2003) Selective upregulation of a single distinctly structured *var* gene in chondroitin sulphate A-adhering *Plasmodium falciparum* involved in pregnancy-associated malaria. Mol Microbiol 49: 179–191.

2. Gardner MJ, Hall N, Fung E, White O, Berriman M, et al. (2002) Genome sequence of the human malaria parasite *Plasmodium falciparum*. Nature 419: 498–511.
