# Supplementary material for: Plasmodium falciparum Variant Surface Antigen Expression Patterns during Malaria
Source: PLoS Pathog. 2005 Nov 18;1(3):e26. doi: 10.1371/journal.ppat.0010026 (PMC1287908; doi:10.1371/journal.ppat.0010026)
Supplement: Supplementary file 6 [file ppat.0010026.sd004.doc]

S3) To test whether a similar conservation was apparent with respect to the regions upstream of the DBLα, reverse primers were designed to a common DNA sequence encoding PoLV1_MFKR_ and PoLV4_PTYF_ which in the 3D7 genome are respectively positively and negatively associated with upsA (P=0.025, P<0.001 respectively, Fishers exact test). These primers were tested against forward primers to upsA and upsB in 5 field isolates. Consistent with these relationships being conserved among the Kilifi *var* genes, product of the correct size were amplified from 4/5 field isolates using the upsA-PoLV1_MFKR_ pair and 4/5 isolates using the upsB-PoLV4_PTYF_ pair but from 0/5 isolates using the upsB-PoLV1_MFKR_ and upsA-PoLV4_PTYF_ pairs.
