# Supplementary material for: Plasmodium falciparum Variant Surface Antigen Expression Patterns during Malaria
Source: PLoS Pathog. 2005 Nov 18;1(3):e26. doi: 10.1371/journal.ppat.0010026 (PMC1287908; doi:10.1371/journal.ppat.0010026)
Supplement: Supplementary file 7 [file ppat.0010026.sd005.doc]

S4) The Mann Whitney U test was used to assess the relationship between the length of DBLα PCR products and each of the 48 different PoLV motifs present in both Kilifi and 3D7 sequences. Only distinct sequences were used in the analysis (see note S1). The results are shown in Table S2. Features indicated in bold in Table S2 were used in a logistic regression analysis to identify those that had a strong independent association with DBLα sequence length. Three degenerate variables, PoLV1_MFK*_, PoLV2_*REY_ and PoLV4_*TNL_ were created as shown in column3 of Table S2. A length cut-off of 117amino acids was chosen since it was the highest value that could split into 2 groups, sequences defined by each sequence feature. The results are shown in Table S3. PoLV2_*REY_ and PoLV1_MFK*_ were selected as having the strongest independent associations with PCR product length and the only features that with a significant independent association with short PCR products. To keep our algorithm simple we chose not to use the PoLV4 motif, though this may be useful in future studies with more sequences.
