# Supplementary material for: Plasmodium falciparum Variant Surface Antigen Expression Patterns during Malaria
Source: PLoS Pathog. 2005 Nov 18;1(3):e26. doi: 10.1371/journal.ppat.0010026 (PMC1287908; doi:10.1371/journal.ppat.0010026)
Supplement: Supplementary file 8 [file ppat.0010026.sd006.doc]

S5) Of the 1746 DBLα sequences obtained from Kilifi, 393 different sequence signatures were identified (see materials and methods and Figure 1C for more information). An additional 46 unique signatures were identified within potentially amplifiable *var* genes from the 3D7 genome. Thus, even after reducing the sequences down to combinations of semi-conserved sequence features, considerable diversity was still evident. Although the majority of sequence signatures (76%) were only identified in single parasite isolates, some were identified in several isolates. Two sequence signatures in particular stand out from this analysis. One signature (called sequence signature 1, “sig1”) was found in every isolate including 3D7, but was never found among the dominantly expressed sequences. A second signature (“sig2”) was found in the dominant sequences from isolates 4140 and 4187, both from children with severe malaria. From isolate 4187, the second most dominant DBLα cDNA sequences also contains sig2. On closer examination these sequences were highly similar to one another (79-90%). See Figure S3 for a more detailed analysis of potentially important sequence signatures.
