# Supplementary material for: Plasmodium falciparum Variant Surface Antigen Expression Patterns during Malaria
Source: PLoS Pathog. 2005 Nov 18;1(3):e26. doi: 10.1371/journal.ppat.0010026 (PMC1287908; doi:10.1371/journal.ppat.0010026)
Supplement: Supplementary file 9 [file ppat.0010026.sd007.doc]

S6) *Var*1 genes are excluded from this analysis. *Var*1 are an unusual class of *var* gene that are expressed constitutively in mature trophozoites [47]. Because *var*1 sequences are expressed mainly in mature trophozoites [47] they are unlikely to have a significant influence on our expression profiles. *Var*1 like sequences were expressed at low levels in some isolates (Figure S3).
