# Supplementary material for: Plasmodium falciparum Variant Surface Antigen Expression Patterns during Malaria
Source: PLoS Pathog. 2005 Nov 18;1(3):e26. doi: 10.1371/journal.ppat.0010026 (PMC1287908; doi:10.1371/journal.ppat.0010026)
Supplement: Supplementary file 10 [file ppat.0010026.st001.doc]

**Table S1** *Parasite isolates and patients used in this study*

|  | DBLα^a^ | | α to β^b^ |  |  | disease |  |  |
| --- | --- | --- | --- | --- | --- | --- | --- | --- |
| Isolate | gDNA | cDNA | gDNA | age | % rosetting^c^ | severity^d^ | VSAab^e^ | ^f^multiplicity |
| 4111 | - | - | 31 | 87 | 47 | S | 0 |  |
| 4013 | 82 | 81 |  | 24 | 22 | S | 2 | 1 |
| 4129 | 78 | 30 |  | - | 5 | M | 1 | 1 |
| 4130 | 22+33^g^ | 73+37 |  | 8 | 6 | M | 0 | 2 |
| 4140 | 80 | 33 |  | 19 | 94 | S | 2 | 3 |
| 4142 | 71 | 41 |  | 40 | 4 | S | 0 | 1 |
| 4161 | 87+36 | 87+36 | 33 | 64 | 0 | S | 2 | 1 |
| 4162 | 72 | 26 |  | 55 | 0 | S | 3 | 1 |
| 4168 | 86 | 73 |  | 85 | 2 | M | 2 | 1 |
| 4172 | 44+30 | 42 |  | 25 | 1 | M | 3 | 1 |
| 4178 | 80+28 | 37+32 |  | 41 | 20 | M | 0 | 1 |
| 4180 | 83 | 31 |  | 4 | 46 | M | 0 | 1 |
| 4187 | 75+37 | 23+40 |  | 84 | 56 | S | 1 | 1 |

^a^ The number of DBLα clones successfully sequenced are shown. ^b^ cloned products from PCR amplification between DBLα and DBL2β. ^c^ The percentage of infected erythrocytes found in close association with at least 2 uninfected erythrocytes. ^d^ S, severe disease; M, mild disease. ^e^ VSA antibody repertoire (the number of 6 parasite isolates recognised by the test plasma). ^f^ Number of genotypes present as determined previously [17]. ^g^ PCR and RT-PCR amplifications for isolates 4130, 4161, 4178 and 4187 were performed in duplicate for both gDNA and cDNA. PCR amplification from genomic DNA was performed in duplicate for 4172. Duplicate estimations of the percentage of cDNA sequences containing 2 cysteines: 4130 (49% and 40% respectively); 4161 (85%, 94%); 4178 (59%, 31%); 4187 (74%, 75%). In all cases except 4178, the dominant cDNA clone had the same sequence. Estimation of cDNA homogeneity (percentage of sequences among the most dominant 2 sequences: 4187 (48% and 53% respectively); 4178 (59%, 38%); 4161 (84%, 94%); 4130 (33%, 32%)
