# Supplementary material for: Plasmodium falciparum Variant Surface Antigen Expression Patterns during Malaria
Source: PLoS Pathog. 2005 Nov 18;1(3):e26. doi: 10.1371/journal.ppat.0010026 (PMC1287908; doi:10.1371/journal.ppat.0010026)
Supplement: Supplementary file 11 [file ppat.0010026.st002.doc]

Table S2. Mann Whitney U test analysis of associations between

sequence features with DBLα sequence length

| feature | z | p | subgroup |
| --- | --- | --- | --- |
| **Cys4** | **-11.3** | **0.0000** |  |
| **PoLV2LRED** | **-13.2** | **0.0000** |  |
| **PoLV3EAIT** | **-4.4** | **0.0000** |  |
| **PoLV4PTYF** | **-12.8** | **0.0000** |  |
| PoLV1LFRG | -3.5 | 0.0005 |  |
| PoLV1LFYG | -3.4 | 0.0006 |  |
| PoLV1LYSG | -3.4 | 0.0007 |  |
| PoLV3KAMT | -3.4 | 0.0008 |  |
| PoLV3EAMT | -2.6 | 0.0087 |  |
| PoLV1LYLG | -2.3 | 0.0223 |  |
| PoLV1LYGG | -2.1 | 0.0365 |  |
| PoLV1LYRG | -1.9 | 0.0527 |  |
| PoLV1LFIG | -1.8 | 0.0680 |  |
| PoLV3KALT | -1.4 | 0.1738 |  |
| PoLV1LYRR | -1.2 | 0.2378 |  |
| PoLV1MYVG | -1.1 | 0.2508 |  |
| PoLV1LFHG | -1.0 | 0.3021 |  |
| PoLV3EALT | -0.6 | 0.5721 |  |
| PoLV1LYEG | -0.4 | 0.6725 |  |
| PoLV4PTKL | -0.4 | 0.7103 |  |
| PoLV3IAMT | -0.4 | 0.7169 |  |
| PoLV3YAIT | -0.1 | 0.8884 |  |
| PoLV4PTNM | 0.6 | 0.5483 |  |
| PoLV4PTNF | 1.0 | 0.2991 |  |
| PoLV2LRDD | 1.2 | 0.2386 |  |
| PoLV2LRNA | 1.3 | 0.2030 |  |
| PoLV3DAIT | 1.4 | 0.1674 |  |
| PoLV3KAIR | 1.4 | 0.1490 |  |
| Cys3 | 1.8 | 0.0716 |  |
| PoLV1LYVG | 1.9 | 0.0574 |  |
| PoLV3KAIT | 2.4 | 0.0175 |  |
| PoLV4PTYL | 2.7 | 0.0063 |  |
| PoLV1LFLG | 3.5 | 0.0004 |  |
| PoLV3RAIT | 3.5 | 0.0004 |  |
| **Cys2** | **11.9** | **0.0000** |  |
| **PoLV2LREA** | **5.7** | **0.0000** |  |
| **PoLV1MFKP** | **4.6** | **0.0000** | PoLV1MFK* |
| **PoLV1MFKR** | **5.1** | **0.0000** | PoLV1MFK* |
| **PoLV1MFKS** | **5.8** | **0.0000** | PoLV1MFK* |
| **PoLV2IREY** | **5.6** | **0.0000** | PoLV2*REY |
| **PoLV2VREY** | **8.3** | **0.0000** | PoLV2*REY |
| **PoLV4LTNL** | **6.3** | **0.0000** | PoLV4*TNL |
| **PoLV4PTNL** | **8.7** | **0.0000** | PoLV4*TNL |

Mann Whitney U test statistic (z) <0 indicates positive associations, z>0 indicates negative associations. P values shown are not adjusted for multiple comparisons.
