# Supplementary material for: Plasmodium falciparum Variant Surface Antigen Expression Patterns during Malaria
Source: PLoS Pathog. 2005 Nov 18;1(3):e26. doi: 10.1371/journal.ppat.0010026 (PMC1287908; doi:10.1371/journal.ppat.0010026)
Supplement: Supplementary file 12 [file ppat.0010026.st003.doc]

Table S3 Logistic regression analysis of associations

between sequence features and DBLα tag sequence length

| feature | Odds ratio  (95% confidence interval) | P |
| --- | --- | --- |
| PoLV4PTYF | 4.78 (2.33-9.80) | 0.000 |
| PoLV2LRED | 3.10 (1.22-7.93) | 0.018 |
| PoLV3EAIT | 1.86 (0.63-5.48) | 0.261 |
| cys4 | 0.90 (0.33-2.41) | 0.827 |
| cys2 | 0.88 (0.29-2.68) | 0.826 |
| PoLV4*TNL | 0.77 (0.34-1.76) | 0.536 |
| PoLV2LREA | 0.58 (0.06-5.36) | 0.628 |
| **PoLV2*REY** | **0.15 (0.06-0.43)** | **0.000** |
| **PoLV1MFK*** | **0.02 (0.003-0.07)** | **0.000** |
